# Evaluation of the Effect of Extracerebral Layers in Cerebral Photoplethysmography (PPG) Using a Head Phantom

**DOI:** 10.3390/s26216987

**Published:** 2026-11-05

**Authors:** Karina Awad-Pérez, Maria Roldan, Panicos A. Kyriacou

**Affiliations:** 1 Research Centre for Biomedical Engineering, City St George’s University of London, London EC1V 0HB, UK; 2 Crainio Limited, London EC3V 9DU, UK

**Keywords:** cerebral photoplethysmography, extracranial circulation, optical brain monitoring, multilayer head phantom

## Abstract

**Highlights:**

**What are the main findings?**

• Extracerebral circulation has a strong contribution to cerebral PPG signals acquired at the forehead, with scalp occlusion producing signal collapse at the proximal detector, while a small residual pulsatile signal (~5% of baseline) remained detectable at the distal detector, indicating sensitivity to brain phantom perfusion at longer source–detector separations.
• Brain occlusion produced a partial amplitude reduction (~45%) with preserved waveform morphology and positive SNR across all wavelengths, demonstrating that cerebral haemodynamic changes are detectable under normal scalp circulation, while also showing a potential masking effect where scalp circulation alone can produce a convincing PPG signal despite complete cerebral hypoperfusion.

**What are the implications of the main findings?**

• Increased source–detector separation (30 mm) improves cerebral signal detection, demonstrating the potential of optical brain monitoring for non-invasive cerebral haemodynamic assessment.
• Conventional forehead PPG configurations are susceptible to extracerebral contamination that may mask cerebral hypoperfusion, highlighting the need for dedicated correction strategies to account for extracerebral contributions in optical brain monitoring.

**Abstract:**

Optical brain monitoring techniques such as near-infrared spectroscopy (NIRS) and cerebral photoplethysmography (PPG) are promising non-invasive approaches for cerebral haemodynamic assessment; however, the measured signal can reflect contributions from both cerebral and extracerebral circulation. This study investigated the effect of extracerebral layers on forehead PPG signals using a multilayer head phantom with independent control of scalp and cerebral perfusion. Three perfusion conditions were performed: both layers perfused, scalp occlusion, and brain occlusion. PPG signals were acquired at 770, 810, and 880 nm using a custom optical probe with proximal (10 mm) and distal (30 mm) source–detector separations. Signal features were extracted and compared across conditions. Scalp occlusion produced a strong reduction in pulsatile signal, with complete signal collapse at the proximal detector. A small pulsatile signal (~5% of baseline amplitude) was detected at the distal detector, indicating that longer source–detector separations (30 mm) can detect signals originating from the brain phantom. Brain occlusion resulted in a partial amplitude reduction (~45%) with preserved waveform morphology across all wavelengths. While this demonstrates that cerebral perfusion changes are detectable in the presence of intact extracerebral circulation, it also highlights a potential masking effect as scalp circulation alone can produce a good-quality PPG signal despite complete cerebral hypoperfusion. These findings have clinical implications and highlight the need for mitigation strategies that account for extracerebral contributions in brain optical monitoring techniques.

## 1. Introduction

Continuous non-invasive monitoring of cerebral haemodynamics has clinical value in conditions like traumatic brain injury, where early detection of cerebral compromise can directly influence patient outcomes [1]. Near-infrared optical techniques, including near-infrared spectroscopy (NIRS), diffuse correlation spectroscopy (DCS), and cerebral photoplethysmography (PPG), have emerged as promising tools for bedside cerebral monitoring due to their non-invasive nature, portability, and potential for continuous real-time measurement [2]. In particular, cerebral PPG acquired at the forehead has attracted growing interest, with recent studies suggesting that forehead PPG signals may contain information related to intracranial pressure (ICP) [3,4,5,6].

However, a fundamental limitation of forehead optical monitoring techniques is that detected photons traverse multiple tissue layers, including the scalp, skull, cerebrospinal fluid (CSF), and brain, before returning to the photodetector. The measured signal therefore reflects contributions from both cerebral and extracerebral tissues and cannot be directly attributed to cerebral haemodynamics alone. It has been suggested that all optical techniques are susceptible to signal contamination from blood flow in the scalp [7], and since both cerebral and superficial layers exhibit pulsatile blood flow waveforms that overlap in time and frequency, their separation represents a challenge [8].

Previous in vivo studies have indicated that optical signals measured at the forehead are strongly influenced by extracranial layers [9,10,11]. However, quantifying these effects in vivo is inherently difficult and relies on approaches such as transient scalp occlusion using head cuffs [8,10]. Head phantoms provide a controlled alternative by replicating anatomical structure, optical properties, and perfusion conditions. Previous phantom studies have investigated extracerebral contamination in optical brain monitoring [12,13,,14,15,16]; however, most have focused on NIRS-derived metrics or device-specific algorithms rather than directly examining light–tissue interactions at the signal level [17]. Controlled in vitro studies specifically assessing the influence of extracerebral layers on cerebral PPG signals have not been performed to date.

In this study, a multilayer head phantom was used to independently control scalp and brain perfusion. The influence of extracerebral layers on forehead PPG signals was investigated by analysing signal amplitude, perfusion index (PI), DC magnitude, and signal-to-noise ratio (SNR) under different perfusion conditions, wavelengths, and source–detector separations. The aim of this work was to quantify the extent to which extracerebral tissues confound PPG measurements and to assess the sensitivity of commonly used wavelengths and source–detector separations to cerebral pulsatility.

## 2. Materials and Methods

### 2.1. Multilayer Head Phantom and Cardiovascular System

**Figure 1:**
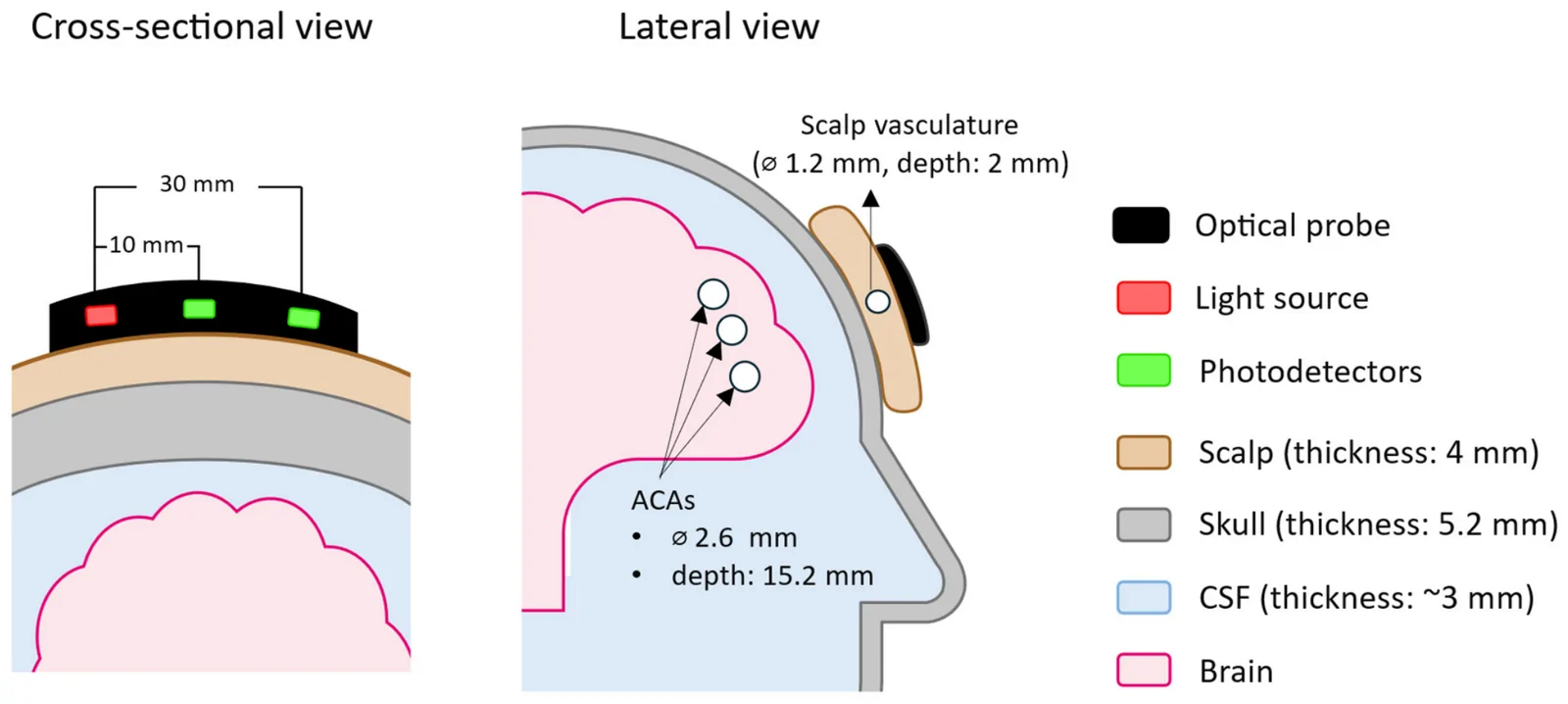
Schematic representation of the multilayer head phantom. Cross-sectional view (**left**) showing the optical probe positioned on the scalp surface, with source–detector separations of 10 mm (proximal) and 30 mm (distal). Lateral view (**right**) illustrating the anatomical layer configuration and vascular dimensions; depth measurements are measured from the phantom surface. ACAs: anterior cerebral arteries; CSF: cerebrospinal fluid; ⌀: diameter.

To evaluate the effect of extracranial circulation on optical signals, a multilayer head phantom was employed [18]. The phantom replicated the anatomical layered structure of the adult human head, with layer thicknesses, vessel dimensions, and optical properties selected to match reported in vivo values [18]. From superficial to deep, the layers consisted of a silicone scalp (4 mm thickness), a 3D-printed rigid skull (5.2 mm thickness), a CSF analogue layer (approximately 3 mm), and a silicone brain model. The scalp vasculature had a vessel diameter of 1.2 mm at a depth of 2 mm, representing the supraorbital and supratrochlear arteries, which are the main vessels of the forehead region sampled in cerebral optical monitoring. Anterior cerebral arteries (ACAs) were represented by custom-made vessels, with a diameter of 2.6 mm at a depth of 15.2 mm from the phantom surface. Figure 1 illustrates the structure of the phantom and Table 1 provides a summary of the optical properties of each layer.

The head phantom was integrated into a closed-loop cardiovascular system driven by a pump (Harvard Apparatus 55-3305; Harvard Apparatus, Holliston, MA, USA) to generate pulsatile flow through the brain and scalp. The pulsatile pump was set to 60 beats per minute with a 40/60 systole-to-diastole ratio and a stroke volume of 20 cc. An optically characterised blood-mimicking fluid was circulated throughout the system [19]. Pressure sensors (Single Use Pressure Sensor; PendoTECH, Princeton, NJ, USA) were used to monitor aortic pressure and ICP. The mean ICP was maintained at approximately 10 mmHg.

The experiment was designed to simulate three physiological conditions:

- Both perfused: Normal perfusion in both scalp and brain.
- Scalp occlusion: normal brain perfusion with scalp hypoperfusion, achieved by occluding flow to the scalp.
- Brain occlusion: normal scalp perfusion with brain hypoperfusion, achieved by occluding flow to the brain.

A control condition where none of the phantoms were perfused was also recorded to confirm the pulsations did not come from residual flow or pump vibration.

**Figure 2:**
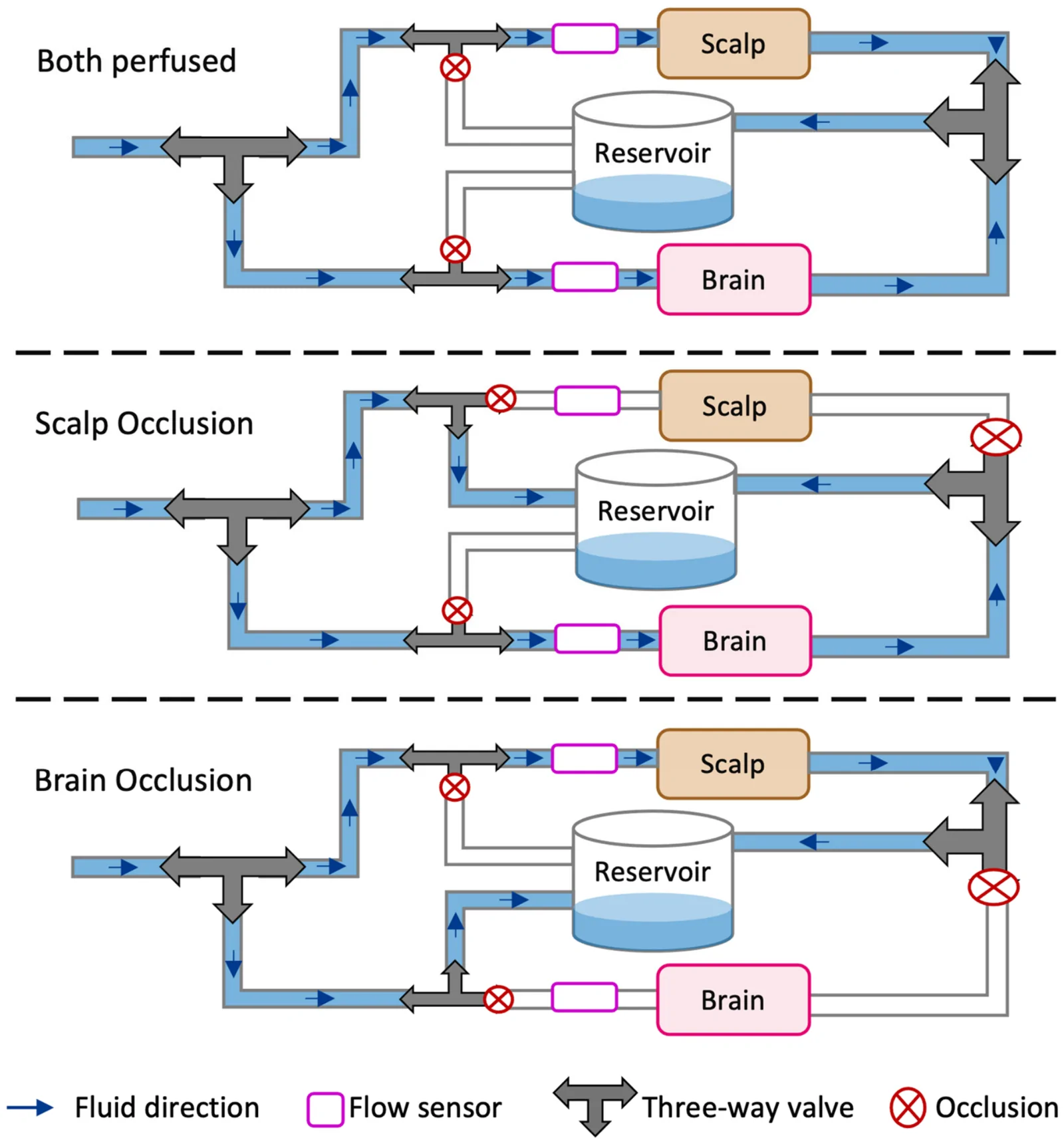
Experimental perfusion conditions. Schematic representation of both phantoms perfused (**top**), scalp occlusion (**middle**), and brain occlusion (**bottom**). Arrows indicate flow direction, purple blocks denote flow sensors, and red symbols indicate occluded flow paths.

The setup enabled selective perfusion of the brain and scalp layers. Three-way valves were used to divert flow and to stop perfusion to either of the phantoms independently. The system was designed such that when flow to one phantom (brain or scalp) was occluded, the diverted volume returned directly to the reservoir via a bypass tube, rather than increasing flow through the other phantom. For example, when flow to the brain phantom was stopped, the excess volume was not redirected to the scalp phantom. This ensured that perfusion in each compartment remained constant regardless of the perfusion state of the other. Flow sensors (SLF3x Series Liquid Flow Sensor; Sensirion, Stäfa, Switzerland) were positioned at the inlet of both the brain and scalp to verify flow conditions under all perfusion states. A schematic of the setup is shown in Figure 2, illustrating the three perfusion conditions.

### 2.2. Signal Acquisition and Analysis

PPG signals were acquired using a custom optical probe and acquisition system (Crainio, London, UK). The probe included light-emitting diodes (LEDs) at 770 nm, 810 nm, and 880 nm, and two square photodetectors positioned at source–detector separations of 10 mm (proximal) and 30 mm (distal), with a proximal-to-distal photosensitive area ratio of 1:14.8. The probe was positioned on the forehead region of the phantom and secured using an elastic band; contact was visually inspected prior to each recording. Signals were amplified with a constant transimpedance gain of 0.4 MΩ across all conditions and replicates, sampled at 200 Hz with a 22-bit ADC resolution, and LED drive current was set to 25 mA for each wavelength. The acquisition system performed an offset calibration to prevent photodetector saturation; raw DC values were reconstructed prior to feature extraction.

Signals were recorded for 5 min under each condition, and the experiment was repeated four times. Between replicates, the blood-mimicking fluid was drained from the system, and the optical probe was removed and placed again on the phantom. Within each replicate, the probe remained in a fixed position, and all perfusion conditions were recorded, such that each occlusion condition was compared against its own baseline.

The PPG signals were first visually inspected to qualitatively assess waveform morphology and pulsatility. Quantitative analysis was then performed to evaluate the influence of extracranial layers on the measured optical signals. The following features were analysed: DC component, SNR, systolic peak amplitude (Asp), and PI.

Each 5-min recording was segmented into non-overlapping 10-s windows. The DC component was obtained by low-pass filtering the raw PPG signal using a third-order Butterworth filter with a cut-off frequency of 0.5 Hz. The magnitude of the DC component was quantified as the absolute value of the low-pass filtered signal, corresponding to the photodiode-detected light intensity.

SNR was computed by separating the pulsatile (AC) component of the PPG signal from high-frequency noise. The AC component was extracted using a fourth-order Butterworth band-pass filter with a passband of 0.4–10 Hz. The noise was estimated by high-pass filtering the signal above 10 Hz using a fourth-order Butterworth filter. SNR was then calculated as the ratio between the mean power of the AC component and the mean power of the noise component, expressed in decibels (dB) (Equation (1)).

(1) $$\mathrm{SNR}=10\times \log_{10}\left(\frac{{\mathrm{AC}}^{2}}{{\mathrm{Noise}}^{2}}\right)$$

Asp was extracted using the pyPPG toolbox (1.0.73) [20], a Python-based (Python 3.10.0) framework designed for standardised PPG biomarker extraction. Asp is defined as the amplitude difference between the pulse onset and the systolic peak within each cardiac cycle. Signal preprocessing and pulse detection followed the standard pipeline implemented in the pyPPG toolbox. Briefly, the signal is band-pass filtered between 0.5 and 12 Hz using a fourth-order Chebyshev Type II filter with a stopband attenuation of 20 dB, followed by 50 ms moving-average filtering. Individual pulse waves are identified by detecting systolic peaks using the Aboy++ algorithm [21], after which pulse onset and offset points are located on either side of each peak to define individual pulse cycles. Fiducial point extraction was verified through visual inspection for all signals prior to feature computation. Asp was computed for each detected pulse and PI was calculated as the ratio between the Asp and DC, expressed as a percentage.

For each replicate, the median of each feature across all windows of the recording was computed; these replicate medians were used as the unit of analysis, and windows were not treated as independent observations. The percentage change between both phantoms perfused (baseline) and each occlusion condition (brain or scalp occlusion) was then calculated within each replicate relative to its own baseline, as shown in Equation (2). This metric provided a normalised measure of the effect of altered perfusion on each feature, for each wavelength and source–detector separation.

(2) $$\mathrm{Percentage change}\left(\%\right)=\frac{\mathrm{Occlusion median}-\mathrm{Both perfused median}}{\mathrm{Both perfused median}}\times 100$$

## 3. Results

### 3.1. Haemodynamic Behaviour Across Perfusion Conditions

Pressure and flow measurements across perfusion conditions demonstrated the expected system behaviour, confirming stability and independent control of scalp and cerebral circulation. Mean aortic pressure remained stable across all perfusion states (49.10 ± 2.12 mmHg). Mean ICP remained approximately constant across all perfusion conditions (10.54 ± 0.22 mmHg). ICP pulsations were present when both phantoms were perfused and during scalp occlusion but disappeared when cerebral perfusion was occluded.

Flow at the ACA inlet remained stable when both phantoms were perfused (49.75 ± 2.66 mL/min) and during scalp occlusion (48.15 ± 2.84 mL/min). When perfusion to the brain phantom was occluded, ACA flow dropped to near zero (0.09 ± 0.04 mL/min) and pulsatile oscillations disappeared, indicating effective interruption of cerebral flow without compensatory redistribution. Similarly, flow at the scalp inlet remained stable when both phantoms were perfused (22.40 ± 1.15 mL/min) and during brain occlusion (21.37 ± 1.16 mL/min). During scalp occlusion, the measured flow decreased to near zero (0.01 ± 0.00 mL/min) and pulsatility was lost, confirming effective isolation of scalp perfusion.

**Figure 3:**
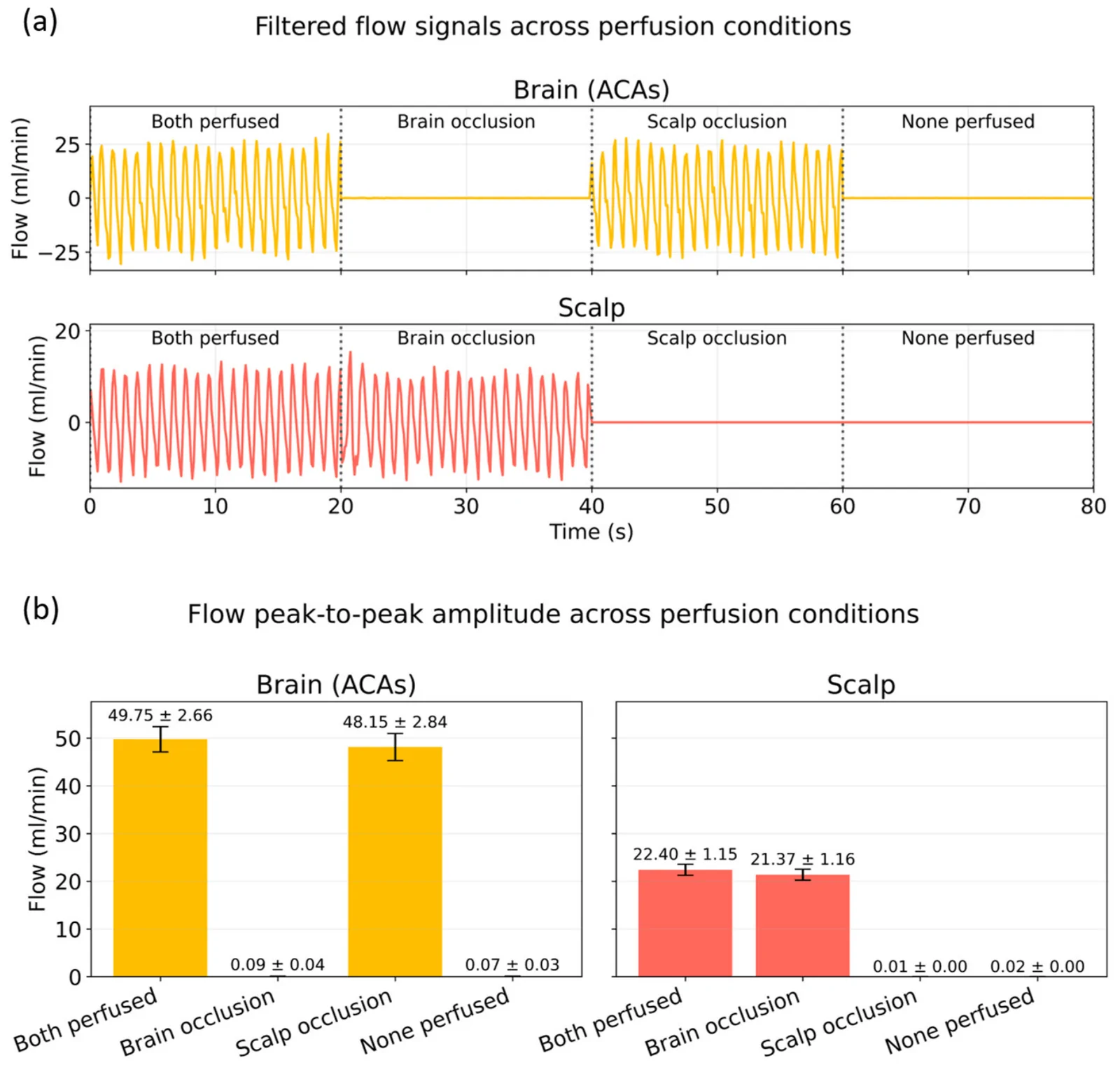
Flow validation across perfusion conditions. (**a**) Representative 20-s segments of filtered flow signals measured at the brain anterior cerebral artery (ACA) inlet and scalp inlet across perfusion conditions. Vertical dashed lines indicate transitions between conditions. (**b**) Peak-to-peak flow amplitude (mean ± standard deviation) for each perfusion condition in the brain and scalp compartments.

Figure 3a shows representative 20-s segments of filtered flow signals from the brain (ACAs) and scalp inlets across the different perfusion conditions, with vertical dashed lines indicating transitions between states. Figure 3b summarises the corresponding peak-to-peak flow amplitudes (mean ± standard deviation) for each condition and phantom.

### 3.2. Effect of Extracranial Circulation

**Figure 4:**
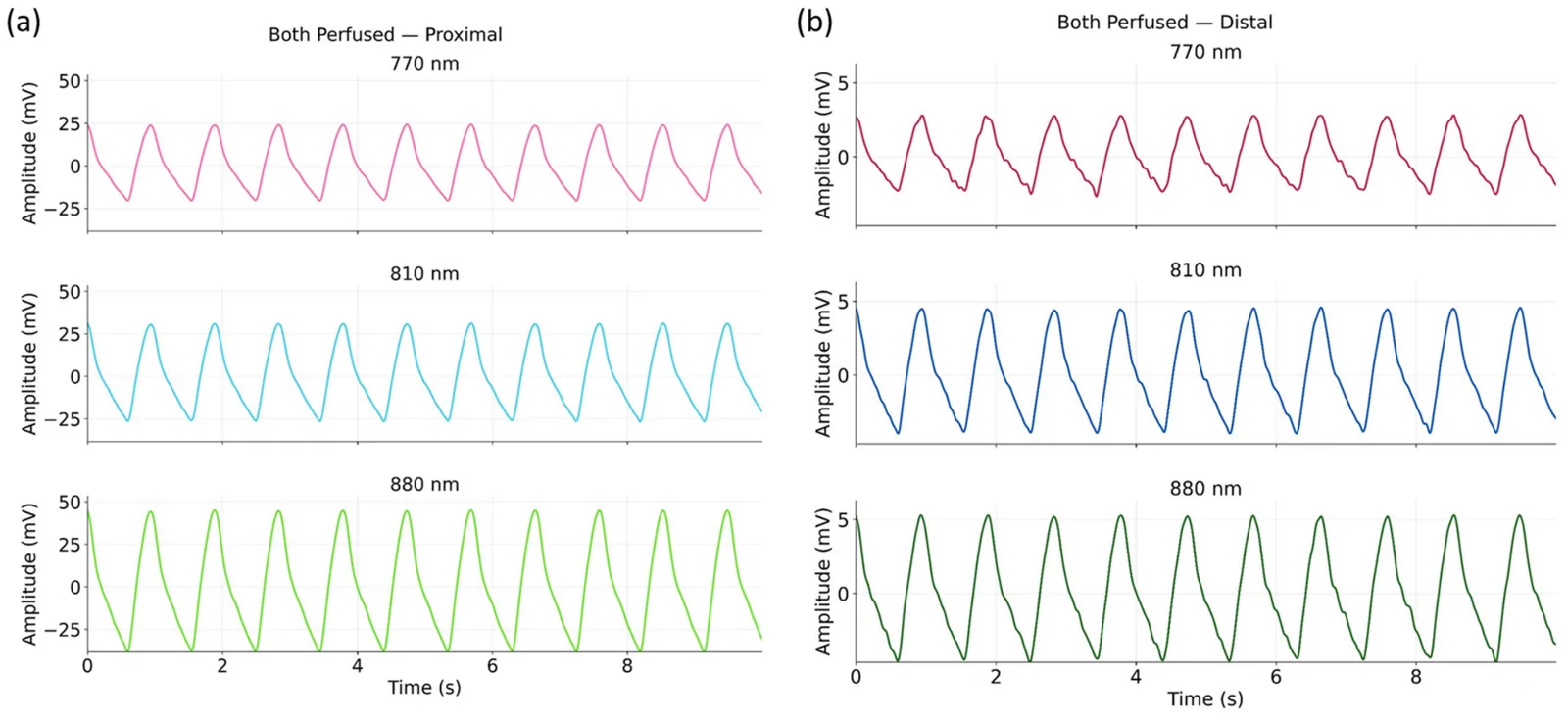
Both perfused (baseline) representative PPG signals. Signals shown for the (**a**) proximal (10 mm) and (**b**) distal (30 mm) source–detector separations. The *y*-axis scale is the same across wavelengths within each source–detector separation but differs between proximal and distal detector.

Under the baseline condition, where both scalp and brain phantoms were simultaneously perfused, clear PPG signals with well-defined morphology were observed across all wavelengths and at both source–detector separations (Figure 4). Features extracted from these baseline recordings are summarised in Table 2.

Asp at the proximal detector was approximately 8–10 times greater than at the distal detector across all wavelengths, and Asp increased with wavelength at both detectors, with the highest values observed at 880 nm. The DC component followed a similar wavelength-dependent trend.

SNR was consistently higher at the proximal than at the distal detector in all replicates, with values of 48.5 (46.2, 49.8) dB at 770 nm, 52.2 (48.2, 53.4) dB at 810 nm and 58.5 (51.3, 59.3) dB at 880 nm at the proximal detector, compared with 28.3 (11.7, 30.3) dB, 40.2 (21.0, 41.2) dB and 41.2 (24.1, 41.9) dB at the distal detector. However, distal SNR values remained high across all wavelengths confirming good signal quality at both source–detector separations under baseline conditions. SNR showed a moderate wavelength-dependent increase, with values rising by approximately 10 dB from 770 nm to 880 nm. However, distal signals at 810 nm and 880 nm showed similar SNR values.

**Figure 5:**
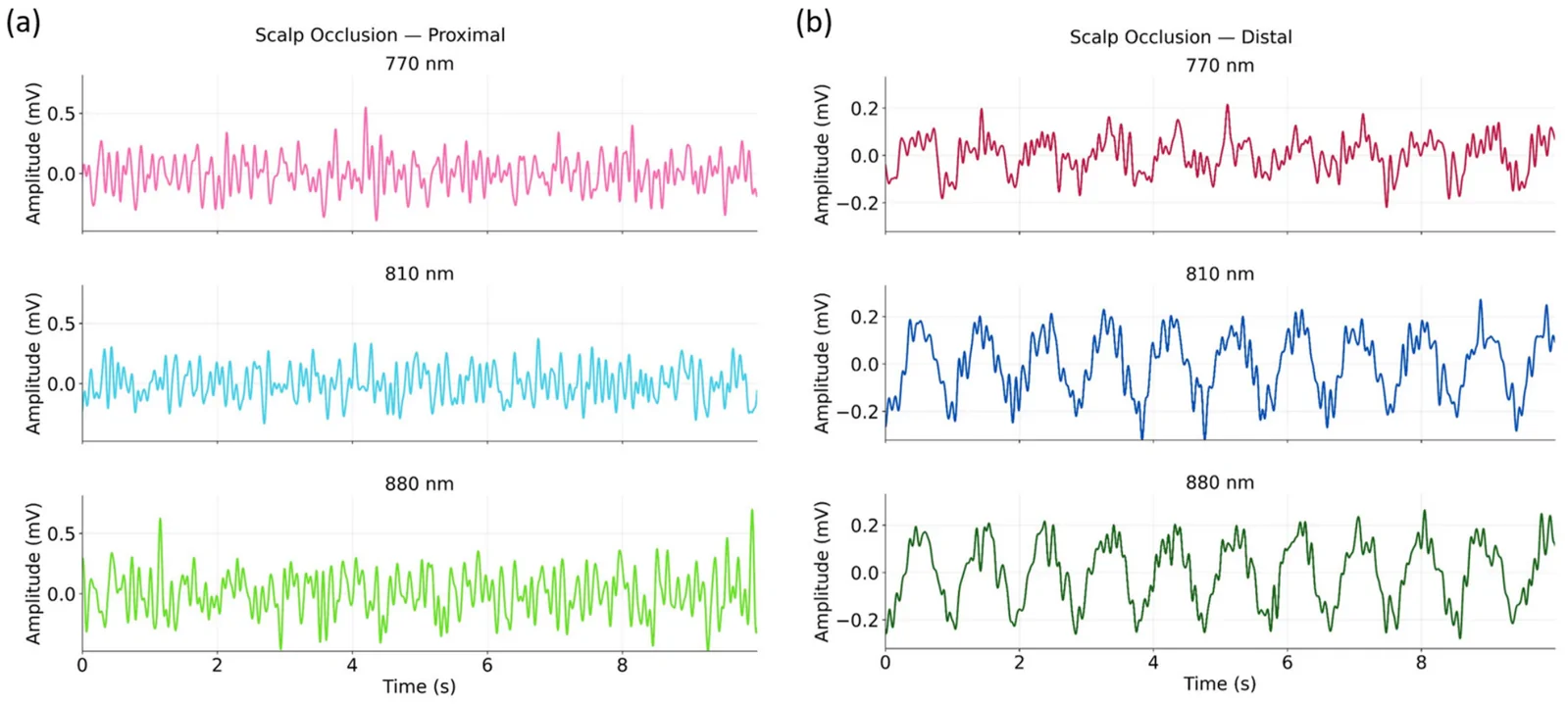
Scalp occlusion representative PPG signals. Signals shown for the (**a**) proximal (10 mm) and (**b**) distal (30 mm) source–detector separations. The *y*-axis scale is the same across wavelengths within each source–detector separation but differs between proximal and distal detector.

When the scalp was occluded, the pulsatile waveform at the proximal detector was completely absent across all wavelengths, and the signal appeared noise-like with no cardiac periodicity (Figure 5a). As a result, no reliable pulses could be identified, and therefore Asp and PI were not extracted for the proximal detector under this condition.

At the distal detector, pulsatile signals remained detectable, particularly at 810 nm and 880 nm (Figure 5b). The waveforms showed reduced amplitude but preserved periodicity consistent with pulsatile pump frequency (60 bpm). The persistence of pulsatile signal at the distal detector during scalp occlusion indicates a residual contribution from the deeper cerebral vasculature within this head phantom model.

**Figure 6:**
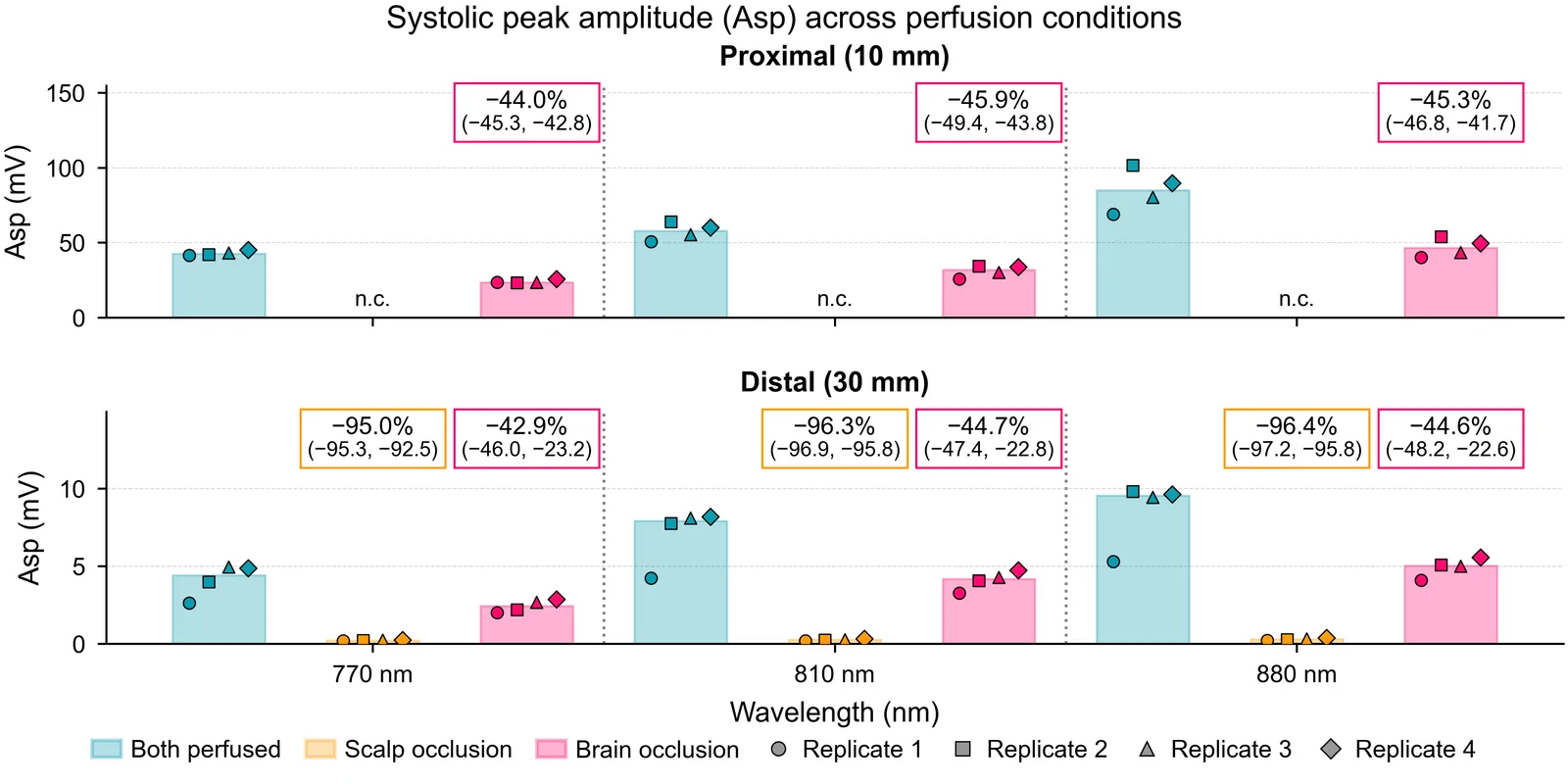
Systolic peak amplitude (Asp) across perfusion conditions for the proximal (10 mm) and distal (30 mm) detectors. Bars show the median across replicates; markers show individual replicates (n = 4). Boxed values show the percentage change relative to both perfused, as median (min, max) across replicates. n.c.: not computed (no detectable pulses).

**Figure 7:**
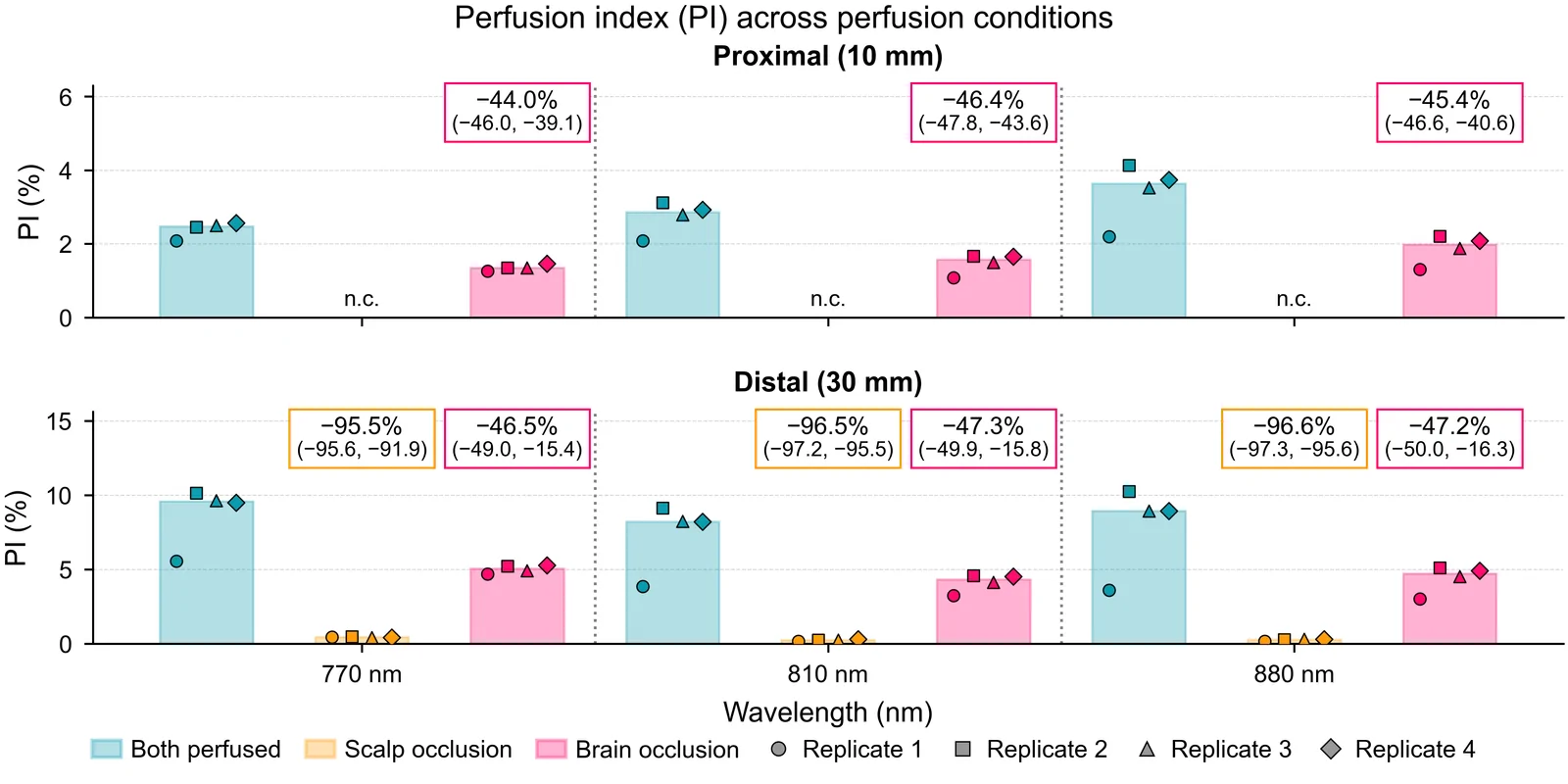
Perfusion index (PI) across perfusion conditions for the proximal (10 mm) and distal (30 mm) detectors. Bars show the median across replicates; markers show individual replicates (n = 4). Boxed values show the percentage change relative to both perfused, as median (min, max) across replicates. n.c.: not computed (no detectable pulses).

Quantitative analysis showed near-complete elimination of pulsatile metrics at the distal detector across all wavelengths (Figure 6 and Figure 7). Asp decreased by 95.0% (92.5, 95.3) at 770 nm, 96.3% (95.8, 96.9) at 810 nm and 96.4% (95.8, 97.2) at 880 nm, with all four replicates decreasing at every wavelength (Figure 6). PI showed an equivalent pattern, with reductions of 95.5% (91.9, 95.6) at 770 nm, 96.5% (95.5, 97.2) at 810 nm and 96.6% (95.6, 97.3) at 880 nm, consistent across all four replicates (Figure 7).

**Figure 8:**
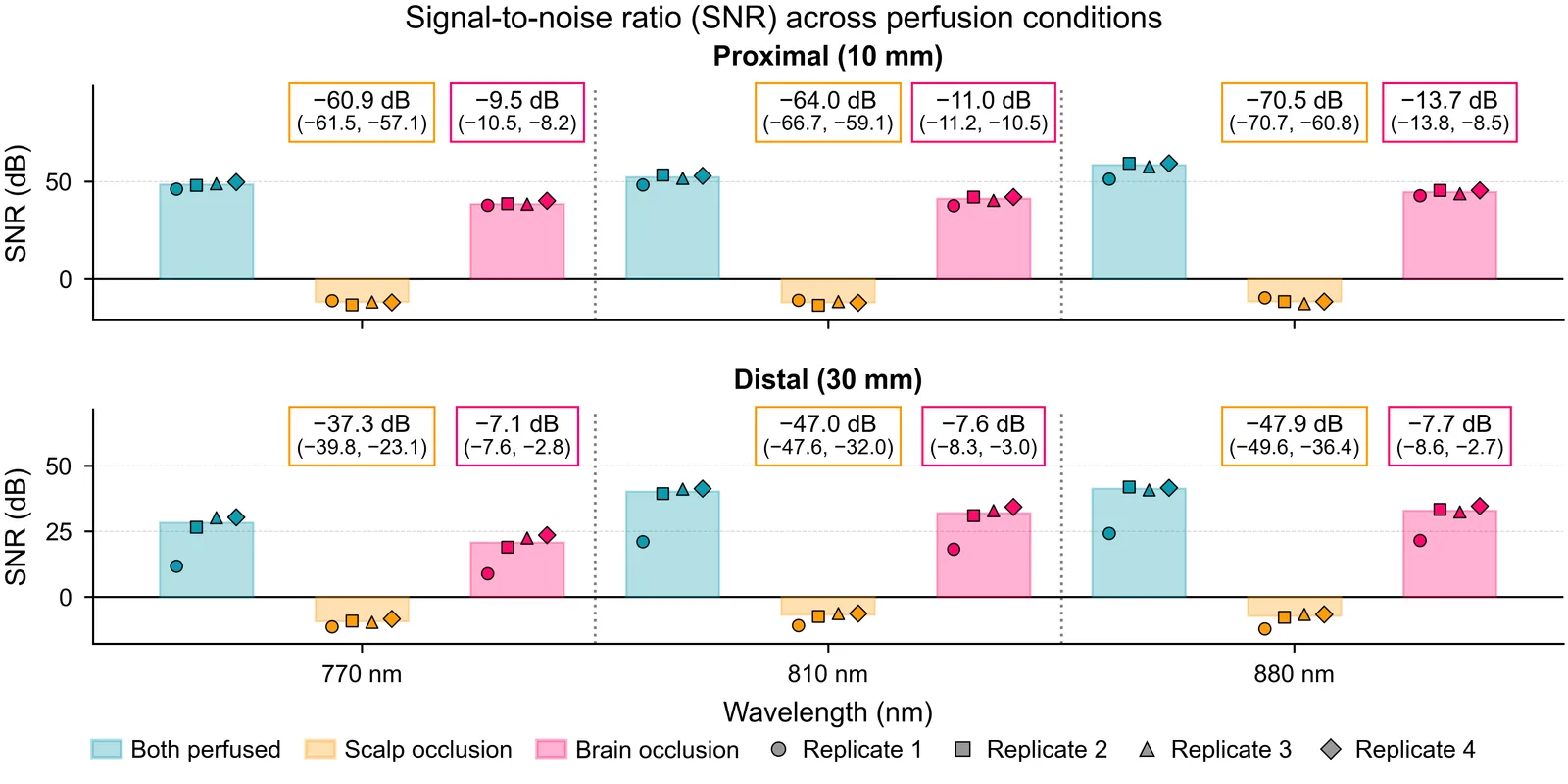
Signal-to-noise ratio (SNR) across perfusion conditions for proximal (10 mm) and distal (30 mm) detectors. Bars show the median across replicates; markers show individual replicates (n = 4). Boxed values show the absolute change (dB) relative to both perfused, as median (min, max) across replicates.

SNR dropped markedly under scalp occlusion, falling below 0 dB across all wavelengths and both detectors (Figure 8). At the proximal detector, SNR decreased by 60.9 dB (57.1, 61.5) at 770 nm, 64.0 dB (59.1, 66.7) at 810 nm and 70.5 dB (60.8, 70.7) at 880 nm. At the distal detector, SNR decreased by 37.3 dB (23.1, 39.8) at 770 nm, 47.0 dB (32.0, 47.6) at 810 nm and 47.9 dB (36.4, 49.6) at 880 nm, with all four replicates decreasing at every wavelength and detector.

The DC component showed no consistent change at the proximal detector, with median changes of +0.9% (−6.3, +1.8) at 770 nm, +0.9% (−2.6, +1.8) at 810 nm and +0.2% (−1.0, +2.0) at 880 nm. At the distal detector, the median DC increased by 8.3% (−8.1, +10.8) at 770 nm, 6.8% (−7.4, +8.2) at 810 nm and 6.6% (−6.2, +8.5) at 880 nm; however, this increase was observed in only three of the four replicates, with one replicate showing a decrease instead. At both detectors, the intervals included zero, and DC changes under scalp occlusion were therefore not considered consistent.

**Figure 9:**
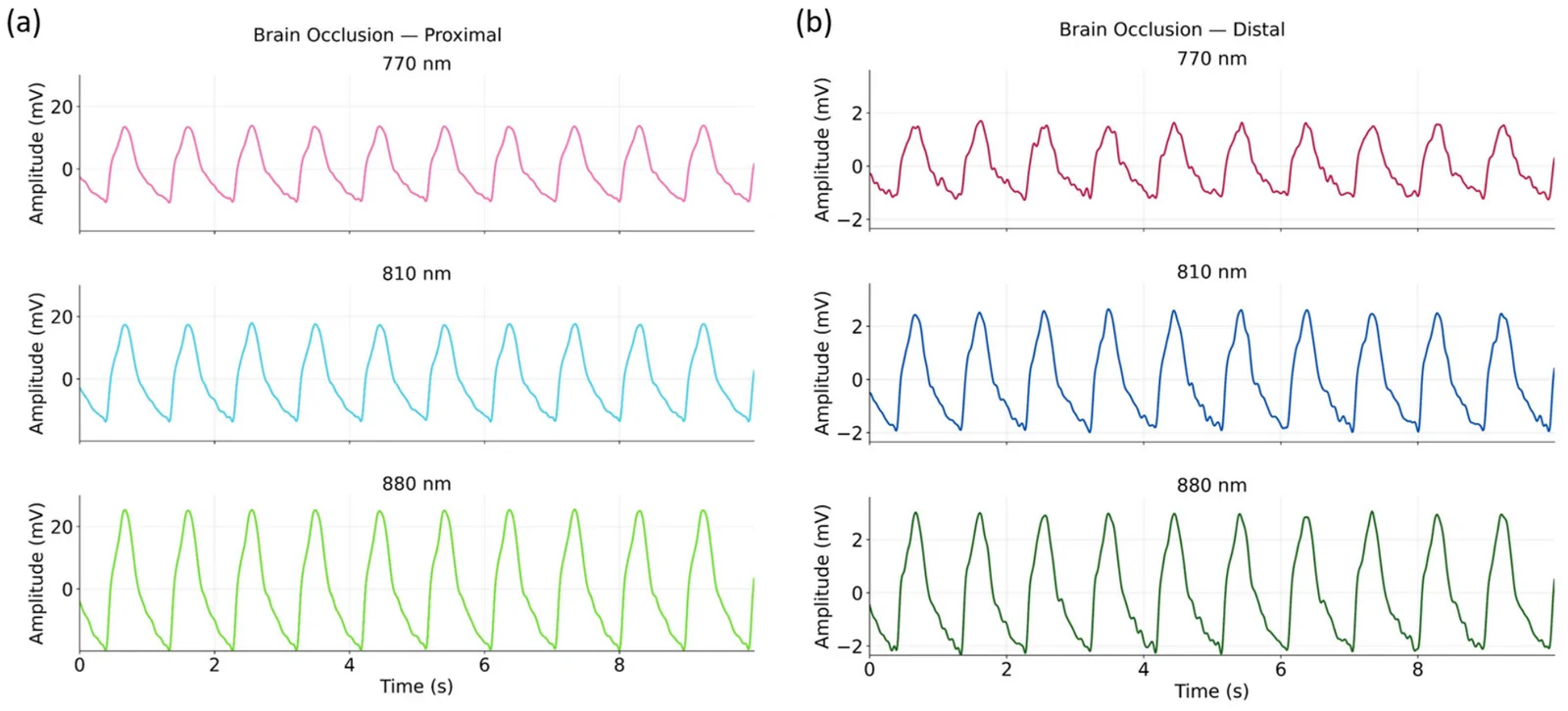
Brain occlusion representative PPG signals. Signals shown for the (**a**) proximal (10 mm) and (**b**) distal (30 mm) source–detector separations. The *y*-axis scale is the same across wavelengths within each source–detector separation but differs between proximal and distal detector.

When the brain phantom was occluded, PPG waveforms remained clearly visible at both proximal and distal detectors across all wavelengths (Figure 9). Unlike scalp occlusion, which caused near-complete signal collapse, brain occlusion preserved well-defined waveform morphology and characteristic periodicity at both source–detector separations, with a partial reduction in amplitude compared to baseline. This confirms that scalp vasculature alone is sufficient to generate a PPG signal even in the complete absence of cerebral perfusion.

Quantitative analysis revealed a consistent decrease in Asp of approximately 45% across all wavelengths and both detectors, with all four replicates decreasing in every case (Figure 6). At the proximal detector, Asp decreased by 44.0% (42.8, 45.3) at 770 nm, 45.9% (43.8, 49.4) at 810 nm and 45.3% (41.7, 46.8) at 880 nm, and at the distal detector by 42.9% (23.2, 46.0), 44.7% (22.8, 47.4) and 44.6% (22.6, 48.2), respectively.

PI showed comparable and consistent reductions across all wavelengths and detectors of 44.0% (39.1, 46.0), 46.4% (43.6, 47.8) and 45.4% (40.6, 46.6) at the proximal detector and 46.5% (15.4, 49.0), 47.3% (15.8, 49.9) and 47.2% (16.3, 50.0) at the distal detector at 770, 810 and 880 nm, respectively (Figure 7).

Similar to scalp occlusion, under brain occlusion the DC component showed no consistent change at the proximal detector, with median changes of +0.3% (−6.8, +1.3) at 770 nm, +0.1% (−3.2, +1.3) at 810 nm and −0.8% (−1.9, +1.5) at 880 nm. At the distal detector, the median DC increased by 6.1% (−9.2, +7.0), 4.9% (−8.4, +5.0) and 4.3% (−7.4, +5.2) at 770, 810 and 880 nm, respectively; however, this increase was observed in only three of the four replicates, with one replicate showing a decrease instead. At both detectors, the intervals included zero, and DC changes under brain occlusion were therefore not considered consistent.

SNR decreased consistently in all four replicates, by 9.5 dB (8.2, 10.5) at 770 nm, 11.0 dB (10.5, 11.2) at 810 nm and 13.7 dB (8.5, 13.8) at 880 nm at the proximal detector, and by 7.1 dB (2.8, 7.6), 7.6 dB (3.0, 8.3) and 7.7 dB (2.7, 8.6) at the distal detector (Figure 8). SNR remained above 0 dB at all wavelengths and both detectors, indicating that signal quality was preserved despite the reduction in pulsatile amplitude. This is in contrast to scalp occlusion, where SNR fell to negative values.

**Figure 10:**
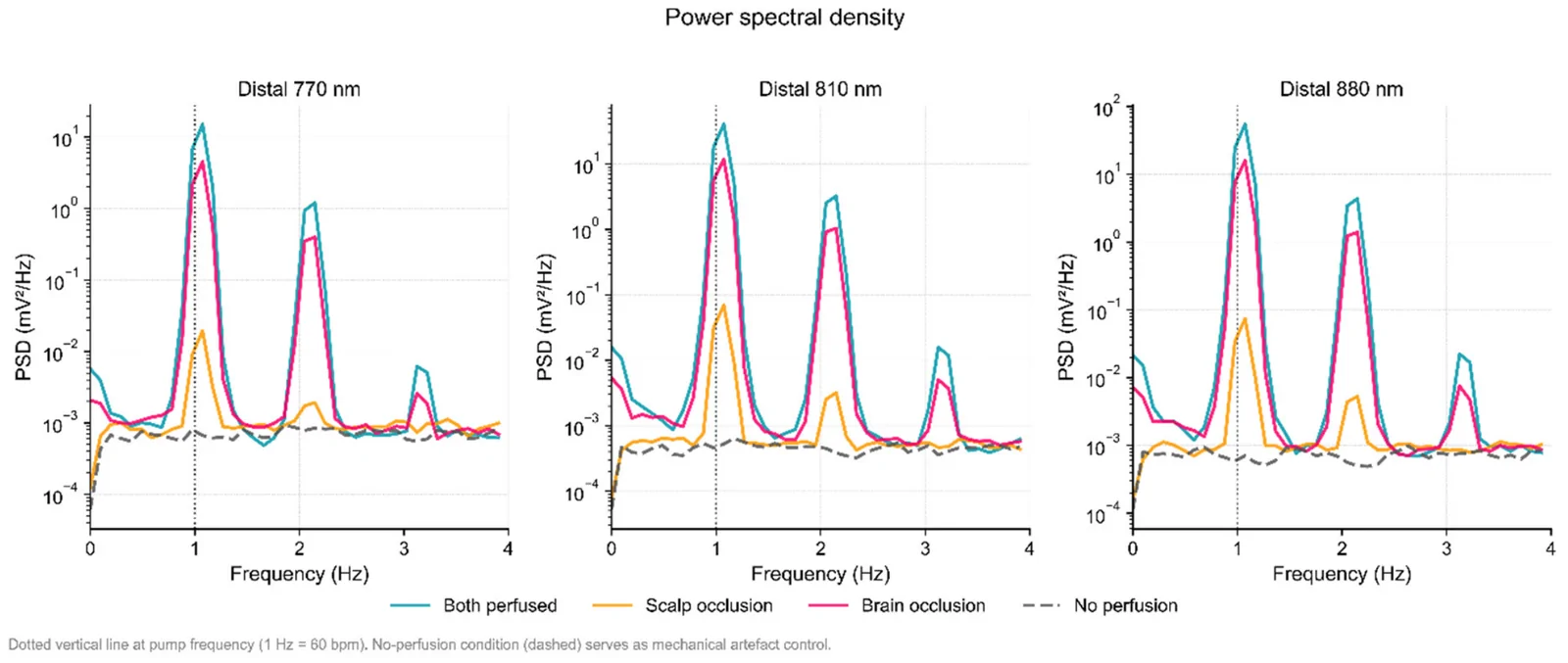
Power spectral density (PSD) of photoplethysmography (PPG) signals at the distal detector across perfusion conditions. The vertical dotted line indicates the pump frequency (1 Hz, 60 bpm). The no-perfusion condition (dashed gray line) shows no spectral peak at any frequency despite the pump remaining active.

Finally, as expected, no pulsatile waveform was observed at either detector under the no-perfusion condition across all wavelengths. This confirms that the pulsatile signals observed under the other conditions originate from volumetric changes within the phantom vasculature rather than from pump-induced mechanical artefacts or residual flow. This was further supported by power spectral density analysis (PSD), which showed no spectral peak at the pump frequency (~1 Hz, 60 bpm) or its harmonics under the no-perfusion condition, whereas a clear peak and corresponding harmonics were present under the other conditions. The PSD at the distal detector across all perfusion conditions is shown in Figure 10.

## 4. Discussion

This study investigated the effect of extracerebral layers on cerebral PPG signals using a multilayer head phantom with independent control of scalp and cerebral perfusion. The experimental setup demonstrated physiologically consistent haemodynamic behaviour across all perfusion conditions. Aortic pressure remained stable throughout (±2.12 mmHg of variation), and selective occlusion of either the scalp or brain phantoms produced the expected flow changes without compensatory redistribution to the non-occluded pathway, confirming independent perfusion control. ICP pulsations were present only during cerebral perfusion and disappeared with brain occlusion, consistent with arterial inflow being a primary driver of ICP pulsatility [22]. This level of experimental control is not achievable in vivo, where scalp and cerebral haemodynamics cannot be independently manipulated. The phantom therefore provides a well-controlled platform for isolating and quantifying the contributions of individual tissue layers to the measured optical signal.

The quality of the PPG signals under the baseline condition confirms the validity of the phantom model. High SNR values indicate good signal quality in PPG measurements [23]. When both layers were perfused, median SNR exceeded 48 dB at the proximal detector and 28 dB at the distal detector across all wavelengths. This confirms that the changes observed under occlusion conditions reflect controlled alterations in perfusion rather than limitations of the acquisition system or head phantom.

The drastic reduction in pulsatile signal under scalp occlusion, with Asp reductions of around 95% at the distal detector and complete signal collapse at the proximal detector, shows that extracerebral circulation dominates the forehead PPG signal. This finding is consistent with previous studies suggesting that forehead perfusion largely dominates optical measurements [10,11], and aligns with Monte Carlo simulations of head tissue optics showing that more than 90% of total optical absorption occurs within the first 16 mm of tissue depth even at source–detector separations of 30 mm [24].

Nonetheless, the distal detector retained a small residual pulsatile signal during scalp occlusion (approximately 5% of the baseline amplitude), indicating that photons reach the cerebral compartment and carry back a detectable pulsatile signal. Although modest, this finding demonstrates that longer source–detector separations (30 mm) offer some detectability of cerebral perfusion, and that brain haemodynamic information can in principle be extracted from distal PPG measurements. Monte Carlo simulations and experimental studies have similarly shown that increasing source–detector separation enhances sensitivity to deeper tissue layers, including the brain [10,11,24,25]. However, both the total number of detected photons and the number of photons reaching the brain decrease exponentially with increasing source–detector separation [8], which is reflected in the lower SNR observed at the distal detector in the present study. It should be noted that this study compared only two source–detector separations (10, 30 mm), and a dedicated multi-separation study would be needed to fully characterise the relationship between separation and cerebral sensitivity.

In addition, at the distal detector during scalp occlusion, the residual pulsatile amplitude increased slightly with wavelength, from 0.22 (0.20, 0.24) mV at 770 nm to 0.25 (0.18, 0.33) mV at 810 nm and 0.30 (0.22, 0.37) mV at 880 nm (Table S2). SNR followed the same trend, being less negative at 810 nm, −6.9 (−11.0, −6.3) dB, and 880 nm, −7.3 (−12.2, −6.7) dB, than at 770 nm, −9.4 (−11.4, −8.4) dB (Table S2). This pattern is consistent with the well-established relationship between wavelength and photon penetration depth in near-infrared tissue optics [26], and with the lower absorption coefficient of the scalp phantom at longer wavelengths, which matches reported optical properties for this tissue layer in humans. However, differences between wavelengths were small and the replicate ranges overlapped, so these observations should be interpreted with caution rather than as definitive evidence of a wavelength advantage for cerebral signal detection.

The complete loss of pulsatile signal and negative SNR at the proximal (10 mm) detector under scalp occlusion confirms that short source–detector separations are predominantly sensitive to superficial tissue. However, the approximately 45% reduction in pulsatile amplitude observed at the proximal detector during brain occlusion indicates that cerebral perfusion changes are detectable in the presence of intact extracerebral circulation.

An important observation from the comparison across perfusion conditions is that the combined PPG signal amplitude cannot be fully explained by a simple linear superposition of independent scalp and cerebral contributions. Under the both-perfused condition, the measured signal amplitude is not equal to the arithmetic sum of the signals obtained during isolated scalp and brain occlusion conditions: at the distal detector, the brain-only signal (scalp occlusion) accounted for 3.6–5.0% and the scalp-only signal (brain occlusion) for 55.3–57.1% of the both-perfused amplitude, together accounting for only approximately 60% of the combined signal in all four replicates. This non-linearity arises because light attenuation depends on both pulsatile and non-pulsatile tissue components [27], and photon path lengths through different layers are not independent; they are dynamically redistributed depending on the absorption and scattering state of all layers. This may have important consequences for signal decomposition algorithms that attempt to remove superficial contributions using simple subtraction or linear regression.

The increases in the DC component at the distal detector observed under occlusion conditions also have implications for NIRS-based cerebral monitoring. The DC component reflects the total light reaching the detector and is determined by attenuation in all tissue layers along the photon path, including the blood in each compartment. Theoretically, a reduction in blood volume in either the scalp or the brain reduces absorption and increases detected light. This effect is expected to be more pronounced at longer source–detector separations, where photons travel longer paths through both superficial and deeper layers. At the distal detector, median DC increased by 8.3%, 6.8% and 6.6% under scalp occlusion and by 6.1%, 4.9% and 4.3% under brain occlusion at 770, 810 and 880 nm, respectively. However, these changes were not consistent across replicates, and their direction and magnitude were similar for two physiologically distinct conditions. This indicates that DC measurements did not distinguish between extracerebral and cerebral perfusion changes. Instead, DC measurements reflect global changes in optical attenuation arising from the combined properties of all tissue layers. As a result, it is not possible to uniquely attribute DC variations to cerebral haemodynamic changes.

Since NIRS devices calculate changes in haemoglobin and oxygenation indices based on NIR light intensity at different wavelengths [28], the non-specificity observed in DC changes would be expected to affect NIRS estimations in the same way. For example, a reduction in scalp perfusion could produce changes similar in direction and magnitude to a true cerebral event, making the two difficult to distinguish.

These findings help explain why extracerebral contamination remains a major challenge for NIRS devices, as has been demonstrated both in vitro [12,13,16] and in vivo [9,10,29,30]. DC measurements are strongly influenced by superficial tissues, such that variations in scalp thickness, blood volume and oxygenation can dominate the recorded signal and mask haemodynamic changes occurring within the brain [31].

Addressing these limitations requires strategies that explicitly account for extracerebral contamination. Approaches such as Monte Carlo-based modelling and correction methods [16], advanced signal processing algorithms [16], multi-distance measurement techniques including spatially resolved spectroscopy (SRS) [28], time-resolved methods incorporating time-of-flight gating [8], or the combination of optical monitoring techniques (hybrid systems) [14] may help mitigate superficial influences. Advancing these strategies will be critical for the clinical translation of optical brain monitoring systems that can reliably monitor cerebral haemodynamics in the presence of extracerebral contamination.

## 5. Limitations and Future Work

This study employed a controlled multilayer head phantom to investigate mechanisms that are challenging to isolate in vivo; however, the model does not fully replicate human physiology. The occlusion conditions represented abrupt, complete interruptions of perfusion to a single compartment, whereas clinical haemodynamic changes are often gradual or partial. The vascular geometry was simplified, and key physiological processes such as cerebral autoregulation and active vascular responses were not reproduced. The findings should therefore be interpreted in the context of the phantom model used.

Skin pigmentation variability was not included, and fixed layer thicknesses do not account for inter-subject anatomical differences. The blood-mimicking fluid did not allow changes in oxygenation, preventing the investigation of scenarios such as cerebral hypoxia in the presence of normal extracerebral oxygenation.

Additionally, fiducial point extraction in signals was verified through visual inspection in this study, an approach that may not be feasible for larger datasets or noisier in vivo recordings. Furthermore, the SNR definition used in this study does not account for low-frequency noise below 0.4 Hz. Applying this approach in vivo may not adequately separate the pulsatile signal from low-frequency interference such as respiration.

Future work could incorporate variability in skin pigmentation and extracranial layer thickness, as well as oxygenation-controlled blood analogues to enable investigation of combined perfusion–oxygenation effects. The head phantom, methodology, and dataset acquired here could be used to evaluate potential correction strategies, including advanced signal processing algorithms, prior to clinical evaluation. Future studies could also investigate additional source–detector separations to better characterise the relationship between separation and cerebral sensitivity, as well as provide additional evidence for the non-linear relationship between scalp and cerebral contributions observed in this study.

## 6. Conclusions

Using a multilayer head phantom that enabled independent control of cerebral and extracerebral perfusion, this study demonstrated a predominant influence of scalp circulation on forehead PPG measurements, particularly at shorter source–detector separations. At longer source–detector separations (30 mm), a residual pulsatile signal originating from the brain phantom was detected during scalp occlusion, indicating that cerebral perfusion contributes a detectable component at this separation; however, this contribution remained small relative to the extracerebral component (approximately 5% of the baseline amplitude). Critically, complete cerebral occlusion preserved PPGs with high SNR, demonstrating that scalp circulation alone can mask the absence of cerebral perfusion. These findings highlight the fundamental challenges of isolating cerebral signals using conventional PPG configurations and underscore the need for approaches that explicitly address extracerebral contamination in optical brain monitoring.

## Figures and Tables

**Table 1:** Optical properties of each phantom layer. μ_a_, absorption coefficient; μ_s_′, reduced scattering coefficient; CSF, cerebrospinal fluid.

Tissue	Wavelength	μ_a_ (mm^−1^)	μ_s_′ (mm^−1^)
Scalp	770 nm	0.0121	1.1183
	810 nm	0.0114	1.0251
	880 nm	0.0096	0.8807
Skull	770 nm	0.0202	0.7428
	810 nm	0.0188	0.7600
	880 nm	0.0189	0.7946
CSF	770 nm	0.00242	0.001
	810 nm	0.00194	0.001
	880 nm	0.00559	0.001
Brain	770 nm	0.0076	1.2667
	810 nm	0.0061	1.3158
	880 nm	0.0109	1.0212

**Table 2:** Both perfused (baseline) PPG features. Values are reported as median (min, max) across replicates for signal amplitude (Asp), perfusion index (PI), signal-to-noise ratio (SNR), and DC component, across wavelengths and source–detector (S-D) separations.

Photodetector (S-D Distance)	Wavelength	Asp (mV)	PI (%)	SNR (dB)	DC (mV)
**Proximal** **(10 mm)**	770 nm	42.50 (41.40, 45.01)	2.48 (2.08, 2.57)	48.5 (46.2, 49.8)	1734.9 (1708.3, 1992.8)
	810 nm	57.67 (50.63, 63.80)	2.86 (2.08, 3.12)	52.2 (48.2, 53.4)	2044.4 (1981.4, 2437.6)
	880 nm	84.88 (68.89, 101.55)	3.63 (2.20, 4.13)	58.5 (51.3, 59.3)	2424.9 (2275.7, 3132.7)
**Distal** **(30 mm)**	770 nm	4.42 (2.62, 4.96)	9.57 (5.56, 10.14)	28.3 (11.7, 30.3)	49.2 (39.3, 51.4)
	810 nm	7.92 (4.23, 8.17)	8.22 (3.85, 9.12)	40.2 (21.0, 41.2)	98.9 (84.9, 109.8)
	880 nm	9.53 (5.30, 9.81)	8.95 (3.59, 10.25)	41.2 (24.1, 41.9)	106.5 (95.7, 147.6)

## Data Availability

The data produced in this study are available upon reasonable request.

## Supplementary Materials

The following supporting information can be downloaded at: https://www.mdpi.com/article/10.3390/s26216987/s1, Table S1: Brain occlusion PPG features; Table S2: Scalp occlusion PPG features.

## References

[1] Roldan Restrepo M. (2022). Non-Invasive Multimodal Monitoring in Traumatic Brain Injury. Ph.D. Thesis.

[2] Karthikeyan P., Moradi S., Ferdinando H., Zhao Z., Myllylä T. (2020). Optics Based Label-Free Techniques and Applications in Brain Monitoring. Appl. Sci..

[3] Dixon B., Sharkey J.M., Teo E.J., Grace S.A., Savage J.S., Udy A., Smith P., Hellerstedt J., Santamaria J.D. (2023). Assessment of a Non-Invasive Brain Pulse Monitor to Measure Intra-Cranial Pressure Following Acute Brain Injury. Med. Devices Evid. Res..

[4] Bradley G.R.E., Kyriacou P.A. (2024). Exploring the Dynamic Relationship: Changes in Photoplethysmography Features Corresponding to Intracranial Pressure Variations. Biomed. Signal Process. Control.

[5] Roldan M., Bradley G.R.E., Mejía-Mejía E., Abay T.Y., Kyriacou P.A. (2023). Non-Invasive Monitoring of Intracranial Pressure Changes: Healthy Volunteers Study. Front. Physiol..

[6] Roldan M., Abay T.Y., Uff C., Kyriacou P.A. (2023). A Pilot Clinical Study to Estimate Intracranial Pressure Utilising Cerebral Photoplethysmograms in Traumatic Brain Injury Patients. Acta Neurochir..

[7] Durduran T., Zhou C., Edlow B.L., Yu G., Choe R., Kim M.N., Cucchiara B.L., Putt M.E., Shah Q., Kasner S.E. (2009). Transcranial Optical Monitoring of Cerebrovascular Hemodynamics in Acute Stroke Patients. Opt. Express.

[8] Hill D.W., Behera A., Etard O., Zhang Y., Tran-Van-Minh A., Ruesch A., Ness L., Maine C., Lashkajani A.S., Parfentyeva V. (2026). Monte Carlo Simulations of Time-Resolved Blood Flow Index: Times-of-Flight beyond ~1 Ns Are Necessary for Brain-Dominated Measurements. Neurophotonics.

[9] Sørensen H., Rasmussen P., Siebenmann C., Zaar M., Hvidtfeldt M., Ogoh S., Sato K., Kohl-Bareis M., Secher N.H., Lundby C. (2015). Extra-Cerebral Oxygenation Influence on near-Infrared-Spectroscopy-Determined Frontal Lobe Oxygenation in Healthy Volunteers: A Comparison between INVOS-4100 and NIRO-200NX. Clin. Physiol. Funct. Imaging.

[10] Davie S.N., Grocott H.P. (2012). Impact of Extracranial Contamination on Regional Cerebral Oxygen Saturation: A Comparison of Three Cerebral Oximetry Technologies. Anesthesiology.

[11] Young A.E.R., Germon T.J., Barnett N.J., Manara A.R., Nelson R.J. (2000). Behaviour of Near-Infrared Light in the Adult Human Head: Implications for Clinical near-Infrared Spectroscopy. Br. J. Anaesth..

[12] Afshari A., Ghassemi P., Lin J., Halprin M., Wang J., Mendoza G., Weininger S., Pfefer T.J. (2019). Cerebral Oximetry Performance Testing with a 3D-Printed Vascular Array Phantom. Biomed. Opt. Express.

[13] Kurth C.D., Thayer W.S. (1999). A Multiwavelength Frequency-Domain near-Infrared Cerebral Oximeter. Phys. Med. Biol..

[14] Verdecchia K., Diop M., Lee A., St. Lawrence K. Characterization of a Hybrid Diffuse Correlation Spectroscopy and Time-Resolved near-Infrared Spectroscopy System for Real-Time Monitoring of Cerebral Blood Flow and Oxygenation. Proceedings of the SPIE.

[15] Verdecchia K., Diop M., Lee A., Morrison L.B., Lee T.-Y., St. Lawrence K. (2016). Assessment of a Multi-Layered Diffuse Correlation Spectroscopy Method for Monitoring Cerebral Blood Flow in Adults. Biomed. Opt. Express.

[16] Forti R.M., Martins G.G., Baker W.B., Mesquita R.C. (2023). Optimizing a Two-Layer Method for Hybrid Diffuse Correlation Spectroscopy and Frequency-Domain Diffuse Optical Spectroscopy Cerebral Measurements in Adults. Neurophotonics.

[17] Awad-Pérez K., Roldan M., Kyriacou P.A. (2025). In Vitro Evaluation of Confounders in Brain Optical Monitoring: A Review. Sensors.

[18] Awad-Pérez K., Roldan M., Kyriacou P.A. (2026). Development and Optical Characterization of a Multilayered Head Phantom for Optical Brain Monitoring. J. Biomed. Opt..

[19] Roldan M., Kyriacou P.A. (2023). Head Phantom for the Acquisition of Pulsatile Optical Signals for Traumatic Brain Injury Monitoring. Photonics.

[20] Goda M.I., Charlton P.H., Behar J.A. (2024). PyPPG: A Python Toolbox for Comprehensive Photoplethysmography Signal Analysis. Physiol. Meas..

[21] Goda M.Á., Charlton P.H., Behar J.A. (2023). Robust Peak Detection for Photoplethysmography Signal Analysis. Proceedings of the 50th Computing in Cardiology Conference.

[22] Ziółkowski A., Kasprowicz M., Kazimierska A., Czosnyka M. (2024). Quantitative Analysis of Similarity between Cerebral Arterial Blood Volume and Intracranial Pressure Pulse Waveforms during Intracranial Pressure Plateau Waves. Brain and Spine.

[23] Charlton P.H., Marozas V., Mejía-Mejía E., Kyriacou P.A., Mant J. (2025). Determinants of Photoplethysmography Signal Quality at the Wrist. PLoS Digit. Health.

[24] Roldan M., Chatterjee S., Kyriacou P.A. Brain Light-Tissue Interaction Modelling: Towards a Non-Invasive Sensor for Traumatic Brain Injury. Proceedings of the 2021 43rd Annual International Conference of the IEEE Engineering in Medicine & Biology Society (EMBC).

[25] Strangman G.E., Li Z., Zhang Q. (2013). Depth Sensitivity and Source-Detector Separations for Near Infrared Spectroscopy Based on the Colin27 Brain Template. PLoS ONE.

[26] Kyriacou P.A., Chatterjee S., Allen J., Kyriacou P. (2022). The Origin of Photoplethysmography. Photoplethysmography.

[27] Chatterjee S., Budidha K., Kyriacou P.A. (2020). Investigating the Origin of Photoplethysmography Using a Multiwavelength Monte Carlo Model. Physiol. Meas..

[28] Shaaban-Ali M., Momeni M., Denault A. (2021). Clinical and Technical Limitations of Cerebral and Somatic Near-Infrared Spectroscopy as an Oxygenation Monitor. J. Cardiothorac. Vasc. Anesth..

[29] Germon T.J., Kane N.M., Manara A.R., Nelson R.J. (1994). Near-Infrared Spectroscopy in Adults: Effects of Extracranial Ischaemia and Intracranial Hypoxia on Estimation of Cerebral Oxygenation. Br. J. Anaesth..

[30] Greenberg S., Murphy G., Shear T., Patel A., Simpson A., Szokol J., Avram M.J., Vender J. (2016). Extracranial Contamination in the INVOS 5100C versus the FORE-SIGHT ELITE Cerebral Oximeter: A Prospective Observational Crossover Study in Volunteers. Can. J. Anesth./J. Can. D’anesthésie.

[31] Roldán M., Kyriacou P.A. (2021). Near-Infrared Spectroscopy (NIRS) in Traumatic Brain Injury (TBI). Sensors.

