# Supplementary material for: Evaluation of the Effect of Extracerebral Layers in Cerebral Photoplethysmography (PPG) Using a Head Phantom
Source: Sensors (Basel). 2026 Nov 5;26(21):6987. doi: 10.3390/s26216987 (PMC13766550; doi:10.3390/s26216987)
Supplement: Supplementary file 1 [file sensors-26-06987-s001.zip › sensors-4611436-supplementary.pdf]

sensors

MDPI

Supplementary material

Table S1. Brain occlusion PPG features. Values are reported as median (min, max) across replicates for signal amplitude (Asp), perfusion index (PI), signal-to-noise ratio (SNR), and DC component, across wavelengths and source–detector (S-D) separations.

Photodetector (S-D distance)	Wavelength	Asp (mV)	PI (%)	SNR (dB)	DC (mV)
Proximal (10 mm)	770 nm	23.51 (23.18, 25.75)	1.35 (1.26, 1.47)	38.6 (37.9, 40.1)	1746.7 (1716.3, 1858.2)
	810 nm	31.91 (25.60, 34.33)	1.58 (1.08, 1.67)	41.2 (37.7, 42.2)	2045.8 (2007.2, 2359.7)
	880 nm	46.43 (40.16, 53.97)	1.98 (1.31, 2.21)	44.7 (42.8, 45.6)	2405.4 (2309.4, 3074.1)
Distal (30 mm)	770 nm	2.44 (2.01, 2.87)	5.07 (4.70, 5.28)	20.7 (8.9, 23.6)	48.6 (42.0, 54.5)
	810 nm	4.17 (3.26, 4.72)	4.33 (3.24, 4.57)	31.9 (18.1, 34.3)	101.9 (89.1, 104.4)
	880 nm	5.04 (4.10, 5.57)	4.72 (3.00, 5.12)	32.8 (21.5, 34.5)	111.9 (99.3, 136.6)

Table S 2. Scalp occlusion PPG features. Values are reported as median (min, max) across replicates for signal amplitude (Asp), perfusion index (PI), signal-to-noise ratio (SNR), and DC component, across wavelengths and source–detector (S-D) separations. n.c.: not computed (no detectable pulses).

Photodetector (S-D distance)	Wavelength	Asp (mV)	PI (%)	SNR (dB)	DC (mV)
Proximal (10 mm)	770 nm	n.c.	n.c.	-11.7 (-13.2, -11.0)	1758.2 (1724.7, 1868.3)
	810 nm	n.c.	n.c.	-11.7 (-13.3, -10.9)	2062.4 (2017.1, 2375.1)
	880 nm	n.c.	n.c.	-11.4 (-12.6, -9.5)	2430.1 (2322.0, 3101.5)
Distal (30 mm)	770 nm	0.22 (0.20, 0.24)	0.44 (0.42, 0.47)	-9.4 (-11.4, -8.4)	49.3 (42.9, 56.7)
	810 nm	0.25 (0.18, 0.33)	0.26 (0.17, 0.31)	-6.9 (-11.0, -6.3)	102.9 (91.5, 107.7)
	880 nm	0.30 (0.22, 0.37)	0.28 (0.16, 0.31)	-7.3 (-12.2, -6.7)	114.2 (102.7, 138.4)
